# Dr. Jacob David Wurtz

**Published:** 1917-10

**Authors:** 


**Dr. Jacob David Wurtz,** Geneva 1867, died at his home in Modena, N. Y., Apr. 26, aged 71. The cause of death was cerebral haemorrhage. He was Clerk of Ulster Co. for 12 years and was a member of the N. Y. legislature for two sessions.

Up to Sept. 18, we have noticed the deaths of 7 members of the Medical Reserve Corps, 3 from disease, apparently before being called to service; 3 from accidental causes while in training camp (gun-shot, collision between auto and trolley, lightning); 1 during active service in France, from an aerial, hostile bomb, apparently deliberately dropped on a hospital.

